# Supplementary material for: Positive Touch Deprivation during the COVID-19 Pandemic: Effects on Anxiety, Stress, and Depression among Italian General Population
Source: Brain Sci. 2023 Mar 24;13(4):540. doi: 10.3390/brainsci13040540 (PMC10136680; doi:10.3390/brainsci13040540)
Supplement: Supplementary file 1 [file brainsci-13-00540-s001.zip › brainsci-2259559-supplementary.pdf]

Supplementary Material for Types of Positive touch deprivation during the COVID-19 Pandemic: Effects on anxiety, stress, and depression among Italian general population

Table S1. Log binomial regression between the prevalence of the four physical positive touch with cohabiting partner and each of the three mental health outcomes (depression, anxiety, and stress).

**DEPRESSION**							
**Variable**	***β***	***SE β***	**Wald's χ^2**	***df***	***p***	***Odds Ratio*** **(e^{β})**	**95%CI (e^{β})**
**How often have you hugged your partner?**	**-1.187**	**0.50**	**5.57**	**1**	**.018**	**0.30**	**0.11 - 0.82**
How often have you kissed your partner?	0.481	0.41	1.36	1	.243	1.62	0.72 - 3.63
How often have you caress your partner?	0.610	0.49	1.57	1	.210	1.84	0.71 - 4.78
How often have you hold your partner's hands?	-0.332	0.34	0.96	1	.327	0.72	0.37 - 1.39
Overall model evaluation: Goodness-of-fit test: Hosmer & Lemeshow: χ^2= 3.13, df= 4, p=.54. Nagelkerke R^2 = .05.							
**ANXIETY**							
**Variable**	***β***	***SE β***	**Wald's χ^2**	***df***	***p***	***Odds Ratio*** **(e^{β})**	**95%CI (e^{β})**
How often have you hugged your partner?	-0.085	0.51	0.03	1	.868	0.92	0.34 - 2.51
How often have you kissed your partner?	0.167	0.44	0.14	1	.705	1.18	0.50 - 2.80
How often have you caress your partner?	-0.666	0.54	1.54	1	.215	0.51	0.18 - 1.47
How often have you hold your partner's hands?	0.136	0.40	0.11	1	.737	1.15	0.52 - 2.54
Overall model evaluation: Goodness-of-fit test: Hosmer & Lemeshow: χ^2= 2.87, df= 3, p=.41. Nagelkerke R^2 = .04.							
**STRESS**							
**Variable**	***β***	***SE β***	**Wald's χ^2**	***df***	***p***	***Odds Ratio*** **(e^{β})**	**95%CI (e^{β})**
How often have you hugged your partner?	-0.477	0.46	1.05	1	.305	0.62	0.25 - 1.54
How often have you kissed your partner?	0.251	0.40	0.40	1	.527	1.28	0.59 - 2.80
How often have you caress your partner?	0.081	0.46	0.03	1	.862	1.08	0.44 - 2.69
How often have you hold your partner's hands?	-0.411	0.33	1.56	1	.211	0.66	0.35 - 1.26
Overall model evaluation: Goodness-of-fit test: Hosmer & Lemeshow: χ^2= 5.08, df= 4, p=.28. Nagelkerke R^2 = .06.							

Table S2. Log binomial regression between the prevalence of the four physical positive touch with cohabiting children and each of the three mental health outcomes (depression, anxiety, and stress).

**DEPRESSION**							
**Variable**	***β***	***SE β***	**Wald's χ^2**	***df***	***p***	***Odds Ratio*** **(e^{β})**	**95%CI (e^{β})**
How often have you hugged your children?	0.541	0.59	0.85	1	.357	1.72	0.54 - 5.42
How often have you kissed your children?	-0.886	0.49	3.21	1	.073	0.41	0.16 - 1.09
How often have you caress your children?	0.032	0.84	0.00	1	.970	1.03	0.20 - 5.37
How often have you hold your children's hands?	0.064	0.65	0.01	1	.922	1.07	0.30 - 3.81

Overall model evaluation: Goodness-of-fit test: Hosmer & Lemeshow: χ^2= 2.85, df= 3, p=.41. Nagelkerke R^2 = .03.

ANXIETY

Variable	*β*	*SE β*	Wald's χ^2	*df*	*p*	*Odds Ratio* (e^{β})	95%CI (e^{β})
How often have you hugged your children?	-0.311	0.67	0.22	1	.641	0.73	0.20 - 2.71
How often have you kissed your children?	0.086	0.55	0.02	1	.875	1.09	0.37 - 3.19
How often have you caress your children?	-0.516	0.99	0.27	1	.602	0.60	0.09 - 4.16
How often have you hold your children's hands?	0.318	0.80	0.16	1	.691	1.37	0.29 - 6.61
Overall model evaluation: Goodness-of-fit test: Hosmer & Lemeshow: χ^2= 3.12, df= 2, p=.21. Nagelkerke R^2 = .03.							

STRESS

Variable	*β*	*SE β*	Wald's χ^2	*df*	*p*	*Odds Ratio* (e^{β})	95%CI (e^{β})
How often have you hugged your children?	0.000	0.58	0.00	1	1.00	1.00	0.32 - 3.13
How often have you kissed your children?	-0.257	0.48	0.28	1	.593	0.77	0.30 - 1.99
How often have you caress your children?	0.986	0.82	1.43	1	.232	2.68	0.53 – 13.50
How often have you hold your children's hands?	-1.142	0.62	3.37	1	.066	0.32	0.09 - 1.08
Overall model evaluation: Goodness-of-fit test: Hosmer & Lemeshow: χ^2= 1.35, df= 2, p=.51. Nagelkerke R^2 = .05.							

Table S3. Log binomial regression between the prevalence of the four physical positive touch with cohabiting relatives and each of the three mental health outcomes (depression, anxiety, and stress).

DEPRESSION

Variable	*β*	*SE β*	Wald's χ^2	*df*	*p*	*Odds Ratio* (e^{β})	95%CI (e^{β})
How often have you hugged your relatives?	-0.288	0.22	1.68	1	.195	0.75	0.48 - 1.16
How often have you kissed your relatives?	0.106	0.22	0.24	1	.624	1.11	0.73 – 1.70
How often have you caress your children?	0.275	0.24	1.26	1	.261	1.32	0.81 – 2.13
How often have you hold your relatives' hands?	-0.096	0.20	0.22	1	.637	0.91	0.61 – 1.35
Overall model evaluation: Goodness-of-fit test: Hosmer & Lemeshow: χ^2= 5.14, df= 6, p=.53. Nagelkerke R^2 = .01.							

ANXIETY

Variable	*β*	*SE β*	Wald's χ^2	*df*	*p*	*Odds Ratio* (e^{β})	95%CI (e^{β})
How often have you hugged your relatives?	-0.304	0.25	1.49	1	.223	0.74	0.45 - 1.20
How often have you kissed your relatives?	-0.31	0.23	1.77	1	.184	0.73	0.46 – 1.16
**How often have you caress your relatives?**	**0.575**	**0.27**	**4.48**	**1**	**.034**	**1.78**	**1.04 – 3.03**
How often have you hold your relatives' hands?	-0.043	0.22	0.04	1	.845	0.96	0.62 – 1.47
Overall model evaluation: Goodness-of-fit test: Hosmer & Lemeshow: χ^2= 1.67, df= 6, p=.95. Nagelkerke R^2 = .02.							

STRESS

Variable	*β*	*SE β*	Wald's χ^2	*df*	*p*	*Odds Ratio* (e^{β})	95%CI (e^{β})

How often have you hugged your relatives?	-0.165	0.22	0.55	1	.459	0.85	0.55 - 1.31
How often have you kissed your relatives?	-0.189	0.22	0.75	1	.387	0.83	0.54 – 1.27
How often have you caress your relatives?	0.27	0.25	1.18	1	.276	1.31	0.80 – 2.13
How often have you hold your relatives' hands?	-0.21	0.20	1.05	1	.306	0.81	0.54 – 1.21
Overall model evaluation: Goodness-of-fit test: Hosmer & Lemeshow: χ^2= 9.51, df= 7, p=.22. Nagelkerke R^2 = .03.							

Table S4. Log binomial regression between the prevalence of the four physical positive touch with cohabiting friends and each of the three mental health outcomes (depression, anxiety, and stress).

DEPRESSION							
**Variable**	***β***	***SE β***	**Wald's χ^2**	***df***	***p***	***Odds Ratio* (*e^β*)**	**95%CI (*e^β*)**
How often have you hugged your friends?	-0.14	0.67	0.04	1	.833	0.87	0.24 – 3.21
How often have you kissed your friends?	0.26	0.69	0.14	1	.708	1.30	0.33 – 5.06
How often have you caress your friends?	0.367	0.76	0.23	1	.628	1.44	0.33 – 6.38
How often have you hold your friends' hands?	0.057	0.66	0.01	1	.931	1.06	0.29 – 3.89
Overall model evaluation: Goodness-of-fit test: Hosmer & Lemeshow: χ^2= 4.78, df= 5, p=.44. Nagelkerke R^2 = .09.							

ANXIETY							
**Variable**	***β***	***SE β***	**Wald's χ^2**	***df***	***p***	***Odds Ratio* (*e^β*)**	**95%CI (*e^β*)**
How often have you hugged your friends?	-0.20	0.70	0.08	1	.776	0.82	0.21 – 3.26
How often have you kissed your friends?	-0.139	0.72	0.04	1	.847	0.87	0.21 – 3.57
How often have you caress your friends?	-0.284	0.79	0.13	1	.718	0.75	0.16 – 3.52
How often have you hold your friends' hands?	0.896	0.72	1.55	1	.213	2.45	0.60 – 10.02
Overall model evaluation: Goodness-of-fit test: Hosmer & Lemeshow: χ^2= 5.38, df= 6, p=.50. Nagelkerke R^2 = .06.							

STRESS							
**Variable**	***β***	***SE β***	**Wald's χ^2**	***df***	***p***	***Odds Ratio* (*e^β*)**	**95%CI (*e^β*)**
How often have you hugged your friends?	0.012	0.70	0.00	1	.986	1.01	0.25 – 4.02
How often have you kissed your friends?	-0.218	0.72	0.09	1	.764	0.80	0.19 – 3.33
How often have you caress your friends?	-0.661	0.86	0.59	1	.441	0.52	0.19 – 2.77
How often have you hold your friends' hands?	1.293	0.81	2.56	1	.110	3.64	0.75 – 17.79
Overall model evaluation: Goodness-of-fit test: Hosmer & Lemeshow: χ^2= 3.17, df= 5, p=.67. Nagelkerke R^2 = .11.							

Table S5. Log binomial regression between the prevalence of the four physical positive touch with not-cohabiting children and each of the three mental health outcomes (depression, anxiety, and stress).

DEPRESSION

Variable	β	*SE* β	Wald's χ^2	*df*	*p*	*Odds Ratio* (e^{β})	95%CI (e^{β})
How often have you hugged your children?	-1.422	1.28	1.23	1	.267	0.24	0.02 -2.96
How often have you kissed your children?	1.263	1.66	0.58	1	.447	3.53	0.14 -91.74
How often have you caress your children?	0.511	1.20	0.18	1	.670	1.67	0.16 – 17.54
How often have you hold your children's hands?	-0.624	1.27	0.24	1	.624	0.54	0.04 – 6.49
Overall model evaluation: Goodness-of-fit test: Hosmer & Lemeshow: χ^2= 7.80, df= 4, p=.09. Nagelkerke R^2 = .03.							

ANXIETY

Variable	β	*SE* β	Wald's χ^2	*df*	*p*	*Odds Ratio* (e^{β})	95%CI (e^{β})
How often have you hugged your children?	0.672	1.17	0.33	1	.565	1.96	0.20 – 19.36
How often have you kissed your children?	-0.182	1.65	0.01	1	.912	0.83	0.03 – 21.18
How often have you caress your children?	0.008	1.23	0.00	1	.995	1.01	0.09 – 11.24
How often have you hold your children's hands?	-0.063	1.40	0.00	1	.964	0.94	0.06 – 14.62
Overall model evaluation: Goodness-of-fit test: Hosmer & Lemeshow: χ^2= 2.65, df= 4, p=.62. Nagelkerke R^2 = .03.							

STRESS

Variable	β	*SE* β	Wald's χ^2	*df*	*p*	*Odds Ratio* (e^{β})	95%CI (e^{β})
How often have you hugged your children?	-1.097	1.43	0.58	1	.444	0.34	0.02 – 5.55
How often have you kissed your children?	1.085	1.95	0.31	1	.577	2.96	0.06 -134.18
How often have you caress your children?	0.479	1.40	0.12	1	.731	1.61	0.10 – 24.93
How often have you hold your children's hands?	-0.474	1.53	0.10	1	.757	0.62	0.03 – 12.60
Overall model evaluation: Goodness-of-fit test: Hosmer & Lemeshow: χ^2= 5.62, df= 4, p=.23. Nagelkerke R^2 = .01.							

Table S6. Log binomial regression between the prevalence of the four physical positive touch with not-cohabiting relatives and each of the three mental health outcomes (depression, anxiety, and stress).

DEPRESSION

Variable	β	*SE* β	Wald's χ^2	*df*	*p*	*Odds Ratio* (e^{β})	95%CI (e^{β})
How often have you hugged your relatives?	0.043	0.24	0.03	1	.861	1.04	0.65 – 1.69
How often have you kissed your relatives?	-0.05	0.28	0.03	1	.857	0.95	0.55 – 1.65
How often have you caress your children?	-0.289	0.30	0.90	1	.343	0.75	0.41 – 1.36
How often have you hold your relatives' hands?	0.072	0.24	0.09	1	.768	1.07	0.67 – 1.73
Overall model evaluation: Goodness-of-fit test: Hosmer & Lemeshow: χ^2= 2.50, df= 5, p=.78. Nagelkerke R^2 = .01.							

ANXIETY							
**Variable**	***β***	***SE β***	**Wald's χ^2**	***df***	***p***	***Odds Ratio* (e^{β})**	**95%CI (e^{β})**
**How often have you hugged your relatives?**	**-0.609**	**0.31**	**3.86**	**1**	**.049**	**0.54**	**0.30 – 1.00**
**How often have you kissed your relatives?**	**0.663**	**0.33**	**4.01**	**1**	**.045**	**1.94**	**1.01 – 3.71**
How often have you caress your children?	-0.33	0.34	0.91	1	.339	0.72	0.37 – 1.41
How often have you hold your relatives' hands?	0.176	0.27	0.42	1	.519	1.19	0.70 – 2.03
Overall model evaluation: Goodness-of-fit test: Hosmer & Lemeshow: χ^2= 1.35, df= 5, p=.93. Nagelkerke R^2 = .01.							

STRESS							
**Variable**	***β***	***SE β***	**Wald's χ^2**	***df***	***p***	***Odds Ratio* (e^{β})**	**95%CI (e^{β})**
How often have you hugged your relatives?	-0.174	0.26	0.46	1	.496	0.84	0.51 – 1.39
How often have you kissed your relatives?	0.091	0.29	0.10	1	.755	1.09	0.62 – 1.94
How often have you caress your children?	-0.232	0.32	0.53	1	.465	0.79	0.42 – 1.48
How often have you hold your relatives' hands?	-0.215	0.26	0.70	1	.404	0.81	0.49 – 1.34
Overall model evaluation: Goodness-of-fit test: Hosmer & Lemeshow: χ^2= 2.92, df= 3, p=.40. Nagelkerke R^2 = .04.							

Table S7. Log binomial regression between the prevalence of the four physical positive touch with not-cohabiting friends and each of the three mental health outcomes (depression, anxiety, and stress).

DEPRESSION							
**Variable**	***β***	***SE β***	**Wald's χ^2**	***df***	***p***	***Odds Ratio* (e^{β})**	**95%CI (e^{β})**
How often have you hugged your friends?	-0.088	0.19	0.21	1	.645	0.92	0.63 – 1.33
How often have you kissed your friends?	-0.343	0.24	1.95	1	.162	0.71	0.44 – 1.15
How often have you caress your friends?	0.01	0.27	0.00	1	.970	1.01	0.59 – 1.72
How often have you hold your friends' hands?	0.247	0.22	1.25	1	.263	1.28	0.83 – 1.97
Overall model evaluation: Goodness-of-fit test: Hosmer & Lemeshow: χ^2= 3.67, df= 4, p=.45. Nagelkerke R^2 = .01.							

ANXIETY							
**Variable**	***β***	***SE β***	**Wald's χ^2**	***df***	***p***	***Odds Ratio* (e^{β})**	**95%CI (e^{β})**
How often have you hugged your friends?	0.108	0.21	0.27	1	.606	1.11	0.74 – 1.68
How often have you kissed your friends?	-0.065	0.27	0.06	1	.808	0.94	0.55 – 1.59
How often have you caress your friends?	0.133	0.31	0.19	1	.663	1.14	0.63 – 2.08
How often have you hold your friends' hands?	-0.102	0.25	0.16	1	.690	0.90	0.55 – 1.49
Overall model evaluation: Goodness-of-fit test: Hosmer & Lemeshow: χ^2= 0.81, df= 4, p=.94. Nagelkerke R^2 = .00.							

STRESS

**Variable**	***β***	***SE β***	**Wald's χ^2**	***df***	***p***	***Odds Ratio* (e^{β})**	**95%CI (e^{β})**
How often have you hugged your friends?	0.171	0.19	0.80	1	.371	1.19	0.82 – 1.73
How often have you kissed your friends?	-0.422	0.25	2.80	1	.095	0.66	0.40 – 1.07
How often have you caress your friends?	-0.088	0.28	0.10	1	.756	0.92	0.53 – 1.59
How often have you hold your friends' hands?	-0.079	0.23	0.12	1	.729	0.92	0.59 – 1.45

Overall model evaluation: Goodness-of-fit test: Hosmer & Lemeshow: χ^2= 3.41, df= 4, p=.49. Nagelkerke R^2 = .03.
